# An Account of the Spontaneous Cure of an Aneurism

**Published:** 1792

**Authors:** R. B. Blagden

**Affiliations:** Surgeon at Petworth in Sussex.


VI. *An Account of the fpontaneous Cure of an Aneurifm. By the Same.*

A Tall, mufcular man, about fifty years of age, was unfortunate enough to have the artery wounded in the opening of the bafilic vein in June, 1788. After a very large quantity of blood had efcaped *per faltum*, a confiderable degree of preffure was made on the orifice, and the blood was ftaid. There was an ecchymofis that extended from the fhoulder to the wrift. A tumour, which had a ftrong pulfation in it, immediately began to be formed, and continued to increafe till it equalled the fize of a common cricket ball, and the limb at the fame time grew (to ufe his own words) painfully lifelefs.

It

It had been told him that nothing but an operation could give a chance of preferving his arm; but as he had formerly lived near Pet-worth, and had been a patient of mine, he chofe to fhow me the cafe before he came to a determination concerning it.

When I faw him, it was about fix months after the accident had happened, and I received from him the above account.

On examination, I found the tumour very nearly as he had defcribed it, fomewhat lefs in-deed than a cricket ball, but hard, and with a ftrong pulfation in it. The arm was fhrunk and cold, and the wrift entirely pulfelefs.

The patient having repeatedly affured me that the tumour had been fomewhat larger, and the pulfation ftill ftronger, I did not hefitate to diffuade him from the operation, at leaft for the prefent.

I recommended the conftant ufe of the flefh brufh only to the whole arm, and defired to fee him regularly every fortnight.

Within a few weeks the arm grew a little warm; in about three months I could perceive a tremulous kind of motion at the wrift; the tumour very gradually diminifhed in fize, its pulfation grew weaker, and the motion at the wrift ftronger in the fame proportion; the hardnefs of the tumour likewife increafed as the tumour itfelf became lefs, fo that when it was diminifhed to the fize of a fmall apple, it was perfectly incompreffible: indeed in this fituation it was fo long ftationary, that I began to doubt whether it would ever vary from it; but within the laft eight or nine months even this extreme hardnefs has gradually abated, and the decreafe gone on, till the tumour is become no larger than a full-fized hazel nut, and not harder than a fteatoma. The pulfe too is now as ftrong in that arm as in the other; the arm itfelf is as large, and the power of ufing it, unlefs on very laborious occafions, (which he is cautioned to avoid) nearly equal *.

It is too evident to admit of any doubt that the circulation is principally carried on in its ufual courfe through the artery, and not through the capillary and anaftamofing branches of it.

This cafe may perhaps be thought to add fome little weight to two of the conclufions

* This account, which was written about three months ago, (when I laft faw the arm) is confirmed by another which I received on the 22d of this month.

drawn drawn by Mr. Ford in his excellent paper * on the ſame ſubject, "that nature is capable of "effecting the cure of aneuriſms ſolely by her "own efforts, and that the cure by nature is a "permanent one:" but although I am unable to form a ſatisfactory idea of the manner in which nature has ſo completely effected the cure in the preſent inſtance, I ſtill hope the caſe may not be altogether unworthy of regard; for I confeſs myſelf of Mr. Watſon's opinion †, that the hiſtory of the diſeaſe is not complete, and that every aneuriſmal tumour will be found to have its peculiarities, and of courſe may afford uſeful information.

*Petworth,*

October 27th, 1791.

* London Medical Journal, Vol. IX. page 155.

† Medical Communications, Vol. I. page 182.

